# Planning future studies based on the conditional power of a meta-analysis

**DOI:** 10.1002/sim.5524

**Published:** 2012-07-11

**Authors:** Verena Roloff, Julian PT Higgins, Alex J Sutton

**Affiliations:** a MRC Biostatistics UnitCambridge, U.K.; b Department of Health Sciences, University of LeicesterLeicester, U.K.

**Keywords:** meta-analysis, power, sample size, evidence-based medicine, random effects, cumulative meta-analysis

## Abstract

Systematic reviews often provide recommendations for further research. When meta-analyses are inconclusive, such recommendations typically argue for further studies to be conducted. However, the nature and amount of future research should depend on the nature and amount of the existing research. We propose a method based on conditional power to make these recommendations more specific. Assuming a random-effects meta-analysis model, we evaluate the influence of the number of additional studies, of their information sizes and of the heterogeneity anticipated among them on the ability of an updated meta-analysis to detect a prespecified effect size. The conditional powers of possible design alternatives can be summarized in a simple graph which can also be the basis for decision making. We use three examples from the *Cochrane Database of Systematic Reviews* to demonstrate our strategy. We demonstrate that if heterogeneity is anticipated, it might not be possible for a single study to reach the desirable power no matter how large it is. Copyright © 2012 John Wiley & Sons, Ltd.

## 1. Introduction

New research studies should be planned and designed by taking into account relevant existing evidence. Such evidence—when arising from numerous, reasonably similar, studies—might be summarized in a meta-analysis. In this paper, we address the use of meta-analysis in the planning of new primary research studies. We address in particular the situation in which a meta-analysis is inconclusive, in the sense that it yields a confidence interval that includes effect sizes with different implications. The ideas are motivated by an argument, put forward by Sutton and colleagues, that meta-analyses increasingly have more influence than individual studies 1, with the implication that new studies might sometimes be designed around their expected impact on a meta-analysis rather than for their results to stand alone. In this context, we would wish to have answers to questions such as how a new study will influence the result of the meta-analysis or how much more information might be needed to make the meta-analysis conclusive. It may be that only a small study is needed to have a good chance of confirming a finding or alternatively that the existing evidence suggests an effect size so small that the probability of obtaining a statistically significant result is minimal, and so new studies are contraindicated.

We concentrate on establishing clinical efficacy of an intervention for a particular outcome. We adopt a frequentist statistical framework and address primarily issues of power to detect a prespecified effect size as statistically significant. This involves fixing an overall information size (or the related sample size) for the meta-analysis. Because some data are already available that will contribute to the final meta-analysis, we focus on *conditional power*, which is the power to detect a specified overall mean effect size given the observed result of the existing meta-analysis. Because the methods that we propose imply that the meta-analysis will be repeated again in the future, they should ideally be applied within a framework that allows for repeated testing without affecting the overall significance level. For the final analysis, we recommend the use of a sequential testing regimen as discussed in a recent paper by Higgins *et al*. 2. The combination of conditional power with principles of sequential analysis essentially produces an adaptive design 3.

Our methods are applicable to two different situations. First, they are relevant to the formulation of conclusions as part of a systematic review of existing evidence. Systematic reviews such as those published in the *Cochrane Database of Systematic Reviews* typically provide recommendations for further research, especially if the result is ambiguous. However, these recommendations are rarely explicit, typically suggesting only that further, high-quality studies are required 4. We believe that these recommendations can, and should, be more specific, for example, by clarifying how many future studies are likely to be needed to reach sufficient power. These implications for research also provide a context for deciding when a systematic review is likely to need to be updated after new studies have emerged. For instance, if the result of the analysis is that to gain sufficient power multiple, large studies are necessary, then updating the meta-analysis after only one small study has been published would not bring the meta-analysis to a conclusion, so an update can wait until more studies have been conducted. In this way, the method can be used to prioritize updating of a systematic review, as has been suggested recently by Sutton and colleagues 5.

Second, our methods can be used when planning a primary research study. Resources for clinical research are limited, and funds and patients should be allocated economically and ethically. Thus, for example, when planning a clinical trial, a reliable and timely decision is required about which intervention is superior, involving as few patients and as few resources as possible. Consulting the existing evidence base is an important prerequisite for the conduct of a new study 6. Indeed, research funders frequently request that primary investigators demonstrate the need for additional studies. For example, the UK Medical Research Council asks researchers applying for clinical trial support to cite a systematic review as evidence of the necessity and the specification of the design of their proposed trial 7. Furthermore, *The Lancet* is one of several journals to require clinical trial authors to include a clear summary of existing evidence in their publication and to illustrate how their trial's findings affect this summary, ideally through direct reference to a systematic review and meta-analysis 8. Besides late phase studies, this method could also be of use moving from late phase II studies into phase III if the study populations are similar enough.

The use of meta-analysis to inform the design of future clinical trials has received relatively little methodological attention. Sutton and colleagues used an elaborate simulation method to pick a sample size depending on the probability that the updated meta-analysis will yield a ‘conclusive’ outcome 1. Wetterslev and colleagues described the use of standard power formula to fix the overall information size needed 9. We propose a method based on conditional power that avoids the need for simulation but takes into account the existing data. Although conditional power should ideally be used within a sequential framework for analysing the evidence, we restrict our attention to conditional power in the current paper, because we believe that it can usefully be applied outside such a framework, in particular in the design of a specific new trial.

We organize the rest of the paper as follows. In Sections 2 and 3, we derive expressions for conditional power based on a standard inverse-variance method for a fixed-effect meta-analysis and a random-effects meta-analysis, respectively. In Section 4, we illustrate the influence of heterogeneity and the number of additional studies on conditional power and in Section 5 we illustrate the methods using a series of examples from the *Cochrane Database of Systematic Reviews*. In Section 6, we discuss the use of a similar approach based on precision rather than power. We end the paper with a discussion of the potential implications and limitations of our methods, including the main differences between our methods and those previously described.

## 2. Conditional power of a fixed-effect meta-analysis

We take a standard inverse-variance weighted average approach to meta-analysis, which is described in full detail in 10. Suppose that *n* studies have already been conducted, and each of these contributes an effect estimate *y*_*i*_, *i* = 1, …, *n*. We assume the variances, *v*_*i*_, of these effect estimates to be known and uncorrelated with the effect estimates. The individual studies are weighted in the meta-analysis according to their inverse variances. Specifically, in a fixed-effect meta-analysis model, the weights of the studies are *w*_*i*_ = /1*v*_*i*_. The overall estimate of the effect size from the existing (old) studies is given by


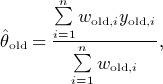


with approximate variance


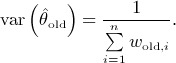


Suppose that a meta-analysis has been conducted, but the result is not conclusive in the sense of having a wide confidence interval that does not exclude either important benefit or lack of effect. One way to analyse the result of a meta-analysis is to calculate its power. Hedges *et al*. 11 described a simple formula to determine the current (unconditional) power of a meta-analysis. Under a standard fixed-effect model, the power of the meta-analysis using a significance level *α* is given by


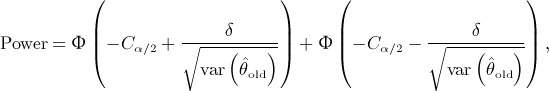
(1)

where *δ* is the effect size under the alternative hypothesis, typically the minimum clinically relevant difference, and *C*_*α*/2_ is the 100 (1 − *α*/2) percentile of the standard normal distribution (i.e. approximately 1.96 for *α* = 0.05) 12. The use of a normal distribution is appropriate when the variances are known, which is a common assumption in meta-analysis that is approximately valid when each study is large. Otherwise, a *t*-distribution may be preferable; but the exact distribution depends on the nature of the effect measure, and for simplicity, we make this large-sample assumption throughout the paper.

If the current power of the meta-analysis is low, then additional information needs to be gathered. Because we have already observed some data, to plan additional trials, it is appropriate to consider conditional power rather than unconditional power, so we calculate the probability of accepting the alternative hypothesis given that the alternative hypothesis is true, conditional on the effect estimate from the existing studies and its uncertainty.

The point estimate from the current meta-analysis is distributed approximately as


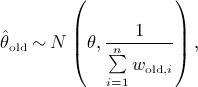


where *θ* is the (assumed common) underlying effect size 13.

Consider now a series of *m* possible future studies, with effect estimates *y*_new.*i*_, *i* = 1, …, *m*. We assume that the future studies are all the same size so that each of the *m* individual new studies has information size *w*_new_, corresponding to the study's weight in a fixed-effect meta-analysis. The estimated effect after the new studies would be


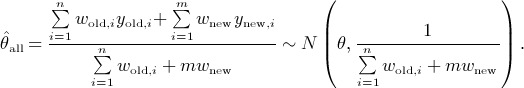


The future effect sizes, *y*_new.*i*_, are unknown. Nevertheless, following Spiegelhalter *et al*. 14, we can construct expressions for conditional power under the fixed-effect meta-analysis model. The result of the meta-analysis will be statistically significant in a two-sided (Wald) test if


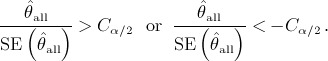


The first corresponds to


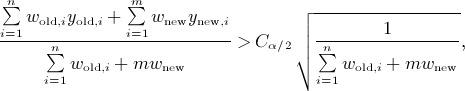


which is equivalent to


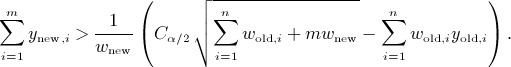


Now under the fixed-effect model and under the specific alternative hypothesis that *θ* = *δ*, we note that 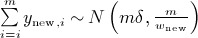, so the probability of the inequality holding is given by


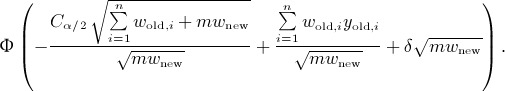


Following an analogous derivation for the probability that 
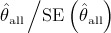
 falls below its critical value, we obtain the two-sided conditional power of the fixed-effect meta-analysis to detect the alternative *θ* = *δ*:


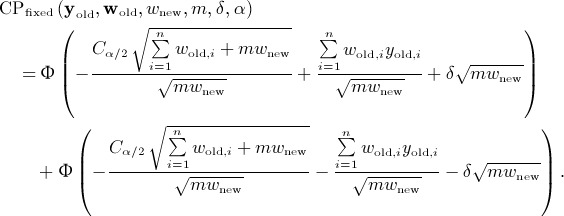
(2)

If a sequential testing regime is applied in the meta-analysis, then the critical value *C*_*α*/2_ should be adjusted in this computation of conditional power in order to give a more appropriate estimate of the necessary sample size.

Note that the conditional power in Equation (2) is a function of the effect estimates from the observed studies *y*_old,*i*_, the weights of the observed studies *w*_old,*i*_, the total information size of the planned studies *mw*_new_, the alternative *δ* and the significance level *α*. Formula (2) is equivalent to the results given by Denne 15, although they expressed theirs in terms of numbers of patients (*n*_1_ and *n*_2_) and among-patient standard deviation (*σ*^2^), so that our future information size, *mw*_new_, is equivalent to their future information size, (*n*_2_ − *n*_1_)/2*σ*^2^. It is also equivalent to the results of Jennison and Turnbull 16, although they expressed theirs using standardized effect estimates rather than unstandardized effect estimates.

## 3. Conditional power of a random-effects meta-analysis

In the random-effects meta-analysis model, we assume that the underlying effect sizes *θ*_*i*_ are normally distributed about a mean *μ* with variance *τ*^2^, where the variance describes the amount of heterogeneity among the studies. The total variance of each study's effect estimate is then the sum of the within-study variance, *v*_*i*_, and the heterogeneity variance, *τ*^2^. The weights awarded to the studies are 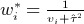, where 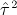 is an estimate of the heterogeneity from the existing studies (e.g. the DerSimonian–Laird estimate). The meta-analysis effect estimate from the existing studies is an estimate of the mean effect


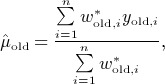


with approximate variance


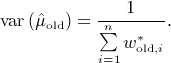


Again, we consider a series of *m* possible future studies, each with within-study information size *w*_new_, but we now need to specify the extent of heterogeneity among the studies’ effect sizes, say, 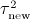. There is no requirement that the same heterogeneity variance should apply to every study. However, in the formulas that follow, for the updated meta-analysis, we will compute a single estimate of the heterogeneity variance, 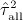, based on the overall heterogeneity among the existing and new studies. The revised weight awarded to an existing study would then be 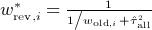, and the weight assigned to a new study would be 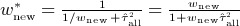. We can write down a general form for the random-effects meta-analysis estimate from all existing and future studies:


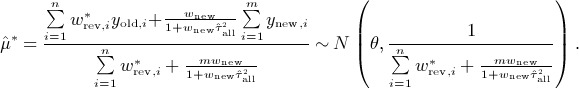


Analogous to the fixed-effect case, we can derive the two-sided conditional power of the extended meta-analysis to detect the specific alternative hypothesis *μ* = *δ* with a critical value *C*_*α*/2_:


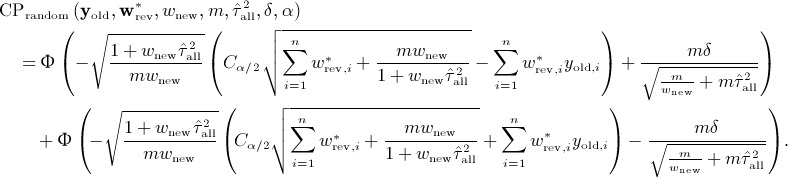
(3)

Note that the conditional power in Equation (3), based on the random-effects model, is a function of the effect estimates from the observed studies *y*_old,*i*_, the weights of the observed studies *w*_old,*i*_, the total information size of the planned studies *mw*_new_, the alternative *δ* and the significance level *α* like the conditional power in the fixed-effect model but, additionally, depends separately on *m*, the number of studies and 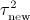 (through 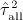), the anticipated heterogeneity among the new studies.

### 3.1 Anticipating the extent of heterogeneity

The random-effects approach requires specification of the extent of future heterogeneity. A simple choice is to assume that future heterogeneity will be the same as in the existing studies. This may be unlikely, however, because we could design all future studies to follow the same protocol with the expected consequence that the 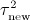 of future studies is lower than the 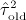 of the existing studies. Alternatively, if we were to decide to look at different subgroups of participants in the different future studies, then we might anticipate that the heterogeneity could be larger than that in the existing studies. Because we plan to use a single overall estimate of the heterogeneity variance in our random-effects meta-analyses, it will prove useful to derive relationships between 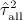, 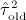 and 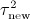. Algebraic relationships among these are not straightforward. We investigated different possibilities on how to produce a good approximation of the overall 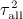 by a weighted average of 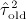 and 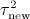. We could either weight by the number of studies *m* and *n* or by the total weight of the studies. In a fixed-effect framework, the total weight of existing studies is 
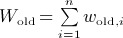
 and that of the planned studies is 
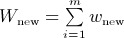
. We define corresponding values for a random-effects framework as 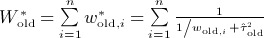 and 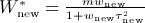. These three options for expressing 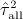 in terms of past and future heterogeneity are then


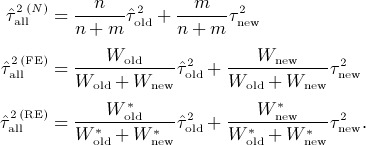


To evaluate the three *ad hoc* estimates, we performed simulations, assuming not only that the weights of the future studies are equal but also that the weights of the existing studies are equal. For the other values, we chose uniformly randomly distributed values for *W*_new_ ∈ (20; 100), *m* ∈ (2; 10), 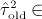 (0.01; 1) and 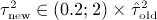. For 10 000 runs, the differences between the directly estimated overall 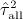 and our three different approximations were 0.065, 0.092 and 0.13 for the aforementioned formulas, respectively. From this, we chose to approximate the overall 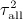 by the weighted average 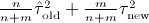.

## 4. Visual exploration of conditional power

By varying the total planned information size, *mw*_new_, the number of planned studies, *m*, and (for a random-effects meta-analysis) the anticipated future heterogeneity, 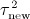, we can determine the conditional power of a meta-analysis to detect a prespecified effect size as statistically significant, under either a fixed-effect model using Equation (2) or a random-effects meta-analysis model using Equation (3). For the remainder of this paper, we shall pursue mainly random-effects meta-analyses, because we consider heterogeneity to be almost inevitable in a meta-analysis. Note that by using simplifying assumptions, we can convert information sizes into approximate sample sizes, as described in our examples in the next section.

To illustrate the conditional power of a meta-analysis under different future research scenarios, we propose the use of two types of graph. The first shows the influence of the number of planned additional studies on the power of a random-effects model, and the second shows the influence of the heterogeneity of the future studies.

To investigate the influence of the number of studies *m*, we keep the heterogeneity of the future studies identical to that estimated from the existing studies; that is, we set 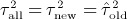. We plot a simple graph that shows how conditional power depends on the additional sample size and on the number of studies. In the following examples, we see that if there is substantial heterogeneity, one big study often cannot give the meta-analysis desirable power.

To investigate the influence of the heterogeneity among the future studies, we use a contour plot. It illustrates possible combinations of sample size and number of studies for which we would expect to obtain a conditional power of 0.9 for different levels of heterogeneity among the future studies. The examples illustrate that, as one would expect, with increasing heterogeneity among the future studies, a larger additional sample size is needed and that for lower heterogeneity, fewer participants are required to reach a certain conditional power. This contour plot also shows conveniently the trade-off between information size and numbers of studies so as to inform a decision between planning more small studies and planning fewer bigger studies.

## 5. Applications

**Figure 1:**
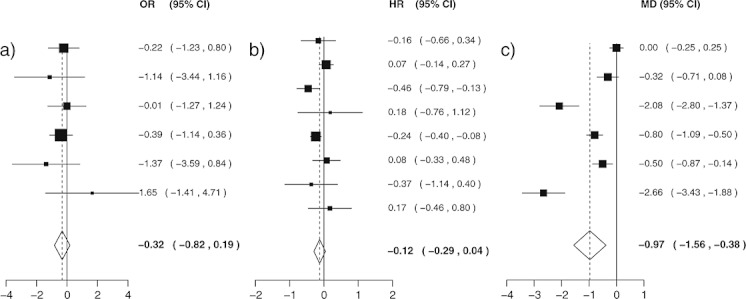
Forest plots of meta-analyses for the examples used in the paper: (a) antibiotic prophylaxis in ear surgery (fixed-effect meta-analysis) 17, (b) preoperative chemotherapy for oesophageal cancer (random-effects meta-analysis) 18 and (c) sublingual immunotherapy for allergic rhinitis (random-effects meta-analysis) 19. OR, odds ratio; HR, hazard ratio; SMD, standardized mean difference.

We apply our method to three examples so as to demonstrate its implications in different scenarios. The first example has binary outcome data, and the existing studies do not demonstrate heterogeneity. We use this to illustrate the fixed-effect version of the method. In the second and third examples, we implement the random-effects version, illustrating application also to survival and continuous outcome data. We provide forest plots for the three examples in Figure 1.

### 5.1 Antibiotic prophylaxis in ear surgery

A Cochrane review of antibiotic prophylaxis in ear surgery included a meta-analysis addressing the binary outcome of postoperative infection within three weeks after surgery 17. The finding is not statistically significant, and the six studies display little heterogeneity (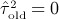, *I*^2^ = 0%). The summary odds ratio (OR) from the six included studies with a total of 1291 participants is 0.73 with a 95% confidence interval from 0.45 to 1.20. The unconditional (retrospective) power of this analysis to detect an alternative OR of 0.61 (equivalent to a log OR of − 0.5) is 49%, using Equation (1).

Our methods are formulated in terms of information size. It is usually more practical to express information requirements in terms of sample size. The approximate variance of a log OR from a single study with two groups each of sample size *k*/2 is


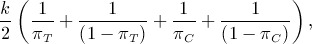


where *π*_*T*_ and *π*_*C*_ are the probabilities of event in the two groups, respectively. By imputing the unknown probabilities, the relationship between the total sample size (*mk*) and the total information size (*mw*_new_) is established. In the implementation that follows, we impute the control group probability by using the arithmetic mean of probabilities across the trials already observed and then apply the alternative hypothesis OR = 0.61 to this to obtain the treatment group probability.

**Figure 2:**
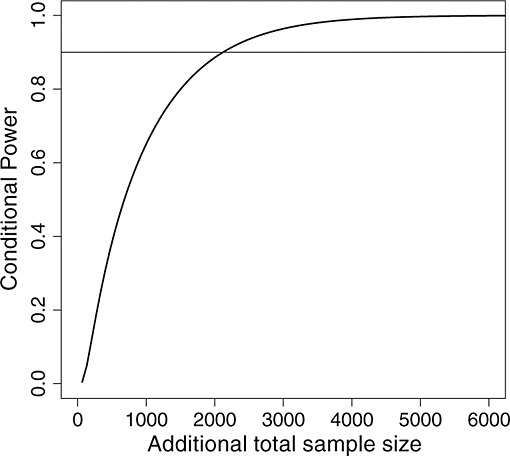
Conditional power of an updated meta-analysis to detect an odds ratio of 0.61, having observed an odds ratio of 0.73 (95% CI 0.44 to 1.2), for a meta-analysis of antibiotics for postoperative infection within three weeks after ear surgery 17, assuming that the additional study does not introduce heterogeneity.

Figure 2 illustrates how the conditional power of the updated meta-analysis depends on the sample size of a single new study, using Equation (2). A desirable power of 90% to detect an alternative of 0.61 can be reached with an additional 1810 patients. For comparison, a single study designed in isolation would require 2610 patients to achieve the same power. Because there is no observed statistical heterogeneity in the studies, the random-effects method would come to the same result. In the absence of heterogeneity, the number of additional studies has no impact on the power.

A possible implication for research in the systematic review might be as follows:

The meta-analysis is not significant in a fixed-effects model, but the current overall treatment effect estimate favours the treatment. If further studies are conducted, a sample size of 1800 patients would give a good prospect of reaching a conclusive result.

### 5.2 Preoperative chemotherapy for oesophageal cancer

To illustrate a random-effects implementation, we take an example from a systematic review of preoperative chemotherapy for oesophageal cancer 18. For the outcome of overall survival, the authors computed a summary hazard ratio (HR) for the comparison of preoperative chemotherapy versus surgery alone by using data from eight studies involving 1729 patients. There was moderate heterogeneity, with *I*^2^ = 40.2%, although the absolute value of 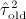 is relatively small at 0.020. The meta-analysis result is not statistically significant, the HR being 0.88 with a 95% confidence interval from 0.75 to 1.04. The unconditional power of the existing studies to detect an alternative HR of 0.82 (equivalent to a log HR of − 0.2) is 64.4%. To express power requirements as a number of events (which is more closely related to information size than sample size), we use the result that the variance of a log HR is approximately equivalent to the number of events divided by four 20.

**Figure 3:**
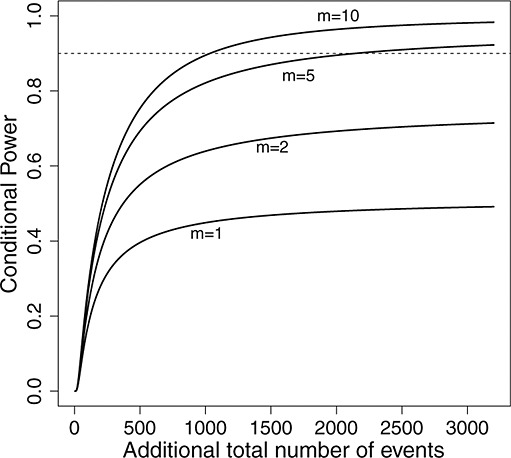
Conditional power of an updated meta-analysis to detect a hazard ratio of 0.82, having observed a hazard ratio of 0.88 (95% CI 0.75 to 1.04), for a meta-analysis of overall survival after preoperative chemotherapy for oesophageal cancer 18, assuming that 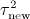 is equal to that in the previous studies. Different curves represent different numbers of future studies (*m*).

Figure 3 illustrates how the conditional power of the updated meta-analysis to detect an HR of 0.82 depends on the number of additional studies, *m*, assuming that 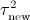 is equal to the observed value in the existing studies, using Equation (3). For *m* = 1, the conditional power does not reach 50% for numbers of events up to 3000. Splitting the information into five equally sized studies with similar heterogeneity increases the power, enabling us to reach a desirable power of 90% with around 2000 events. Splitting the information further up into *m* = 10 studies enables us to reach 90% conditional power with only 1000 events, again assuming that the heterogeneity of the future studies is similar to that of the observed ones.

**Figure 4:**
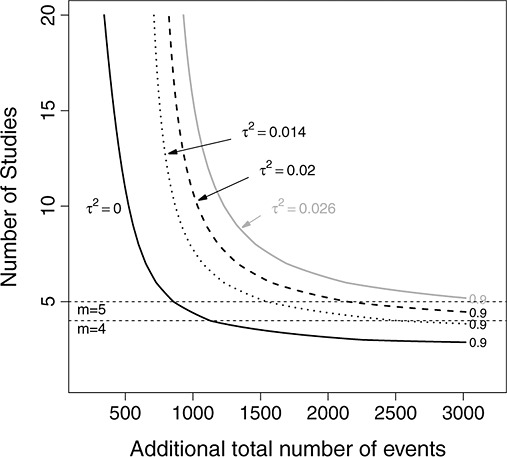
Contours for a conditional power of 90% for an updated meta-analysis to detect a hazard ratio of 0.82, having observed a hazard ratio of 0.88 (95% CI 0.75 to 1.04], based on a meta-analysis of overall survival after preoperative chemotherapy versus surgery for oesophageal cancer 18. The graph illustrates the relationship between additional information size (expressed as number of additional events) and number of planned studies for four values of future heterogeneity 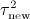.

Figure 4 shows the contour plot of possible combinations of additional events and number of studies for which we would expect a conditional power of 90% for the meta-analysis. The contours show clearly the trade-off between additional information size and number of studies. To reach a specific power, either a fixed amount of information would need to be split into several studies or the size of each of a fixed number of studies would need to be increased. Here, economic, logistic and ethical arguments need to be considered to decide if it is better to conduct more studies with fewer patients or fewer studies with more patients. As would be expected, with a higher 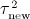, either more information or more studies are needed, and with lower 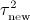, either less information or fewer studies are needed. The impact of 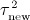 on the power is not substantial across the range plotted, owing to the moderate size of the observed heterogeneity. So to reach a conditional power of 90% with 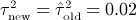, we would need 2000 events split into *m* = 5 additional studies.

We also investigated the implications for power of taking one large study and arbitrarily splitting it into multiple sub-studies without heterogeneity, that is, with 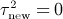. The conditional power on adding a single study with 2000 events is 55%, breaking it into two homogenous studies of 1000 events each increases power to 79%, and breaking it into four studies of 500 events each increases power to 95%. This seemingly inappropriate gain in power highlights the need for our methods to be used with integrity, a point to which we return in the discussion.

A possible implication for research in the systematic review might be as follows:

The random-effects meta-analysis is not statistically significant, but the current overall treatment effect estimate does not rule out a beneficial effect of treatment. Therefore, the authors recommend that further studies are conducted. Owing to the moderate heterogeneity already observed, multiple studies are likely to be needed to allow a firm conclusion to be drawn. For example, if similar heterogeneity is to be observed in the future, then five studies with 2000 events in total should provide 90% power.

### 5.3 Sublingual immunotherapy

To illustrate implications of substantial heterogeneity on conditional power, we take a meta-analysis from a systematic review of sublingual immunotherapy for allergic rhinitis 19. The finding for allergic rhinitis symptom scores for allergies caused by house dust mites, expressed as a standardized mean difference (SMD), is not statistically significant ( SMD = − 0.58 with a 95% confidence interval from − 1.43 to 0.27). So far, there are only 229 patients in six studies included in the meta-analysis, and the unconditional power of these studies to detect an alternative SMD of − 0.5 is 20%. Hence, further studies could be necessary. The heterogeneity is substantial, with *I*^2^ = 88% and 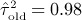. To translate from information size to sample size, we use the approximate variance of an SMD from a single study as 4 / *k*, again assuming a total sample size of *k* for each study and a randomization ratio of 1:1.

**Figure 5:**
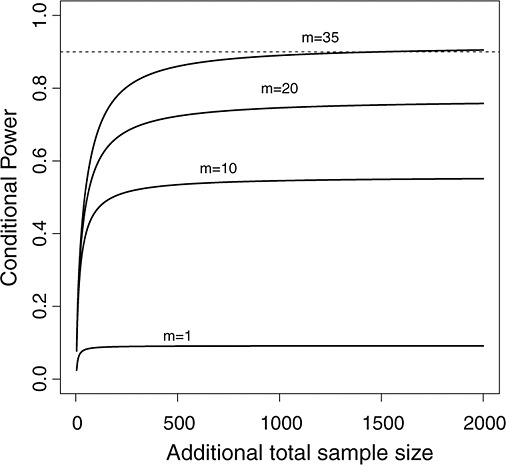
Conditional power of an updated meta-analysis to detect a standardized mean difference of − 0.5, having observed a standardized mean difference of − 0.58 (95% CI − 1.43 to 0.27), for a meta-analysis of allergic rhinitis symptom scores after sublingual immunotherapy for allergic rhinitis caused by house dust mites 19, assuming that 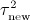 is equal to that in the previous studies. Different curves represent different numbers of future studies (*m*).

Figure 5 shows the conditional power for different combinations of additional sample size and number of studies. It reveals that with one large study, we cannot reach a conditional power of 90% to detect even a moderate SMD of − 0.5, assuming that future heterogeneity is the same as the observed heterogeneity. Splitting the same information into 10 studies with similar heterogeneity gives a conditional power of only 60%. To reach a desirable conditional power of 90%, we would need to conduct 35 additional studies with an overall number of 1250 participants.

**Figure 6:**
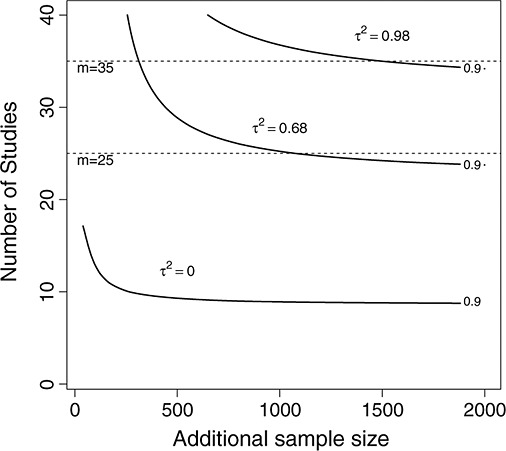
Contours for conditional power of 90% for an updated meta-analysis to detect a standardized mean difference of − 0.5, having observed a standardized mean difference of − 0.58 (95% CI − 1.43 to 0.27), based on a meta-analysis of allergic rhinitis symptom scores after sublingual immunotherapy for allergic rhinitis caused by house dust mites 19. The graph illustrates the relationship between additional information size (expressed as sample size) and number of planned studies for three values of future heterogeneity, 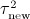.

Figure 6 provides the contour plot illustrating combinations of additional sample size and number of studies, which produce a conditional power of 90%. A drop of 30% in the heterogeneity variance among the future studies (to 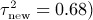 has a significant impact on the number of studies needed: we would then be able to reach a conditional power of 90% with 25 studies instead of 35. The example illustrates that the anticipated extent of heterogeneity in the future studies is a strong determinant of the number of studies needed to achieve a specific power. In this example, however, even if no heterogeneity was expected in the future, multiple studies would still be necessary to reach the desirable conditional power

A possible implication for research in the systematic review might be as follows:

The meta-analysis is not statistically significant, the heterogeneity among the existing studies is substantial and the strength of evidence in favour of a clinically relevant difference is weak. Even with multiple large studies, the probability of detecting a difference in the meta-analysis is very low. Therefore, the authors recommend that further studies of the same question should not be undertaken.

## 6. Precision

An alternative to planning a series of additional trials based on conditional power is to plan them with regard to the precision of the meta-analytic estimate. For example, to conclude that two interventions have similar effects, a confidence interval should lie within a range of equivalence. We define precision as the inverse of the variance, and it reflects directly the information size or total weight awarded to studies in the meta-analysis. In a random-effects model, the precision of the updated meta-analysis is given by


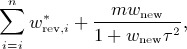


where we would use an updated estimate of the heterogeneity variance in the revised weights, 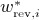. One way to illustrate the influence of the number of future trials, *m*, on the precision of the estimate is to plot *m* against the width of the updated confidence interval expressed as a proportion of the width of the original confidence interval.

This proportion is given by


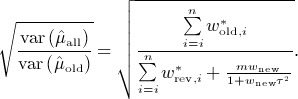


**Figure 7:**
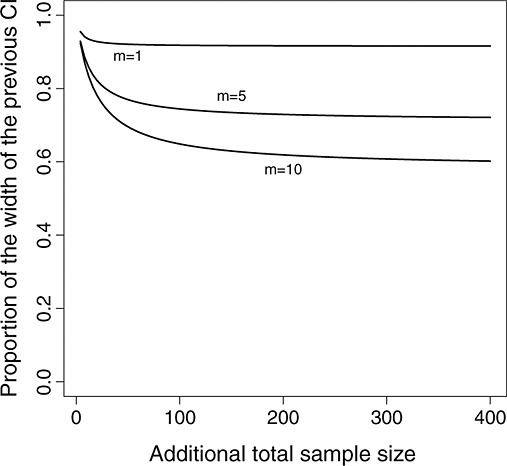
Precision of an updated meta-analysis (expressed as relative reduction in confidence interval width) for a meta-analysis of allergic rhinitis symptom scores after sublingual immunotherapy for allergic rhinitis caused by house dust mites 19, assuming that 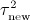 is equal to that in the previous studies. Different curves represent different numbers of future studies (*m*).

We illustrate such a plot for the sublingual immunotherapy example in Figure 7, in which we assume that the heterogeneity of future studies is similar to that among the six observed studies. Because the heterogeneity, with *I*^2^ = 88% and 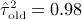, is substantial for this example, adding a single study of up to 400 participants does not reduce the confidence interval width by more than 8%, and distributing these participants among 10 studies fails to reduce the confidence interval width by more than 39%.

## 7. Discussion

When a meta-analysis is inconclusive, it often points to a need for further research. Planning of such further research should take into account the findings of the meta-analysis. Meta-analyses may be more influential than individual studies, for example, because healthcare policy makers often base decisions on systematic reviews of reliable evidence rather than on single clinical trials. It may therefore be sufficient for the planning of new research to focus on whether the meta-analysis is likely to become conclusive rather than whether the new research is conclusive in isolation. In this paper, we propose a method that uses the conditional power of an updated meta-analysis to inform recommendations for further research. These recommendations may be used either in drawing conclusions from systematic reviews or to support the design of new studies by primary researchers wishing to influence the result of the meta-analysis.

Our methods are similar in spirit to those proposed by Sutton *et al*. 1. The authors describe an approach based on assurance 21, the expected impact on the meta-analysis of more studies that are exchangeable with the studies already observed. Our approach follows the more classical tradition of evaluating conditional power to detect a specific alternative hypothesis. Sutton *et al*. implemented their approach by using MCMC methods, although simpler approximations could be made. Our approach leads naturally to simple formulas that can be implemented easily; a computer spreadsheet implementation of our formulas is available from the authors. An alternative method proposed by Wetterslev *et al*. 9 uses a basic fixed sample size formula to determine a needed information size to detect a given alternative. The variance is estimated from the observed studies, but the method does not take into account the observed treatment effect.

An important aspect of the use of power calculations is the specification of a clinically important effect to act as an alternative hypothesis. Such important effect sizes are rarely made explicit in systematic reviews, although their use has been proposed to assess reliability and conclusiveness of a meta-analysis 22. Appropriate effect sizes may be available from one or more of the individual study reports in the review, although these may be strongly dependent on resources available for running the study. Review authors should choose these values carefully, considering any more recent developments in the field. It may be appropriate to investigate power for a variety of different alternative hypotheses.

On applying our conditional power formulas to a variety of examples of inconclusive meta-analyses, we demonstrate that in a random-effects model, conducting one additional study often might be insufficient to yield a statistically significant result, no matter how large the study. This happens only when there is heterogeneity among the studies, for example, owing to different doses or age groups of participants. Depending on the magnitude of this heterogeneity, multiple studies might be indicated to reduce uncertainty around the mean sufficiently to produce a conclusive result. Does this suggest that we should split up existing data into different data sets? We demonstrated in our second example that breaking a study into homogenous sub-studies can achieve a gain in power. The extent of this gain will depend on the relative numbers of old and new studies. For instance, if there are similar numbers of old and new studies of similar sizes, then the revised estimate of heterogeneity variance will be approximately halved on adding the homogenous new studies. This observation depends on the way in which heterogeneity variances are estimated for the totality of trials. In the approach that we described, we computed a single revised heterogeneity variance based on the combined old and new trials, because this reflects standard practice in updating meta-analyses. An alternative approach would be to use 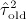 in the weights for the existing studies and 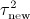 in the weights for the new studies. Note that breaking multicentre trials into their component centres might produce more legitimate increases in power if the variation in the locations, populations and implementations of interventions across centres mirrors such variation across different trials. This would also improve estimation of the between-study variance, which is poorly estimated when there are few studies.

In planning future trials, it is unlikely that a series of multiple studies will be undertaken solely because of observed heterogeneity among the existing studies. Our findings in this regard may therefore have primary use in determining when an update to a systematic review is unlikely to lead to any changes in conclusions. This would be the case when there is substantial heterogeneity among the observed studies, such that a very large number of future studies would be required, no matter how similar their results, to reach high power in an updated random-effects meta-analysis.

Updating a meta-analysis with an additional study (or studies) creates a multiple testing scenario, which standard meta-analysis methods would ignore. To ensure the correct type I error rate in the final analysis combining old and new studies, nominal significance levels need to be adjusted to preserve the overall type I error rate. Several authors have proposed sequential approaches to meta-analysis #b^2^b^23^b^24^b^25^, and although these approaches do not lead directly to recommendations on planning future studies, they can be used in combination with our method to control the overall type I error rate at the final analysis. Adaptive design methods for clinical trials provide a convenient framework in which to achieve this #b^26^b^27^b^28^. We have, for example, implemented the method of Bauer and Köhne, in which conditional power is used to determine the number of additionally needed studies, and Fisher's combination rule for *p*-values is used to combine the original with the new studies 26. Under a fixed-effect model, the two sources of evidence are independent, and the method can be applied directly. For the random-effects model, however, the use of a common estimate of *τ*^2^ causes the *p*-values from the two stages to be dependent, inflating the type I error. In subsequent works, we are investigating the practical implications of this.

A further technical limitation is that our methods are based on information size rather than on sample size, and we make approximate transformations between the two based on properties of the observed studies (e.g. control group risk or among-person standard deviation). The assumptions that we make to perform these transformations might turn out to be wrong, although such assumptions are involved in the majority of standard sample size calculations.

A practical limitation of the use of conditional power calculations is the need for future studies to be sufficiently similar to the existing studies for a meta-analysis to be meaningful. There are many situations in which this may not be the case. For example, the underlying standard of care may improve over time, yielding a diminishing treatment effect (or ‘placebo creep’) 29. If studies are performed as a direct consequence of a finding of an earlier study, then the assumed lack of dependence between studies is broken. One particular problem that permeates many meta-analyses is the possibility that subsequent studies are performed because of a statistically significant finding in an early study. This may lead to a ‘regression to the mean’ phenomenon, such that subsequent studies fail to replicate the result 30. Such dependence across studies impacts on the validity of meta-analyses, although the impact may be small once the number of studies reaches a reasonable size, and meta-analyses of small numbers of studies must always be treated with considerable caution.

An alternative approach to assessing the value of further research is through the use of value of information methods #b^31^b^32^. This has been proposed by economists as a rational way to decide whether the benefits of improved decision making, through the reduction in uncertainty in the parameter(s) in a decision model, outweigh the monetary cost of collecting the necessary data. Such an approach can be very computationally demanding, and estimation of required parameters (such as the lifespan of an intervention) can be hard. However, it does provide a rational framework and avoids making decisions based on arbitrary thresholds of statistical significance so, thus, will probably be preferred in an economic decision context.

In conclusion, the aim of this paper had been to present a simple approach to using meta-analysis to aid the planning of future trials. Computations of conditional power are straightforward and lead to informative graphical illustrations of possible scenarios. We propose that systematic review authors and clinical trial planners should consider these approaches to examine the potential impact of future studies on the results of meta-analyses in a more quantitative manner than has hitherto been done.
